# Will a plant germplasm accession conserved in a genebank change genetically over time?

**DOI:** 10.3389/fpls.2024.1437541

**Published:** 2024-10-03

**Authors:** Yong-Bi Fu

**Affiliations:** Saskatoon Research and Development Centre, Agriculture and Agri-Food Canada, Saskatoon, SK, Canada

**Keywords:** plant genetic resource, long-term germplasm conservation, genebank management, germplasm regeneration, genetic change

## Abstract

The simplified question on the genetic change of a conserved plant germplasm accession over time is raised for a better understanding of the challenging mission of conserving more than 7.4 million germplasm accessions in 2000 genebanks worldwide for generations to come. Its answer will influence how these genebanks operate to ensure the continued survival and availability of the conserved plant genetic resources for future food security. Here, we explore the expected impact of evolutionary forces on plant germplasm in genebanks, search for the theoretical expectations and empirical evidence for such impacts from the literature, and discuss the ramifications of the evidence for long-term plant germplasm management and conservation. It is expected that genetic changes of long-term conserved germplasm under genebank conditions will occur commonly as an evolutionary rule, not as an exception. Incorporating evolutionary biology into the Genebank Standards and operational procedures will benefit the mission of long-term germplasm conservation.

## 1 Introduction

“The only constant in life is change.” - Heraclitus

The ancient Greek philosopher Heraclitus, quoted above, recognizes the essential, underlying essence of life as change. That perspective is also relevant, and perhaps still instructive, for us to understand the complexity and challenges in the mission of the long-term conservation of plant genetic resources to underpin present and future food security for humanity (Plucknett, 1987; Gepts, 2023). During the last 60 years, plant germplasm conservation efforts globally have achieved much progress toward attaining this goal (Harlan, 1975; Frankel, 1987; Pistorius, 1997; Gepts, 2006; Engels and Ebert, 2021a). Over 7.4 million plant germplasm accessions are currently conserved in 2000 genebanks worldwide (FAO, 2010; Engels and Ebert, 2024). The International Treaty on Plant Genetic Resources for Food and Agriculture (Esquinas-Alcázar, 2005) to conserve and utilize these plant genetic resources was established in 2004 (Gepts, 2006; FAO, 2010) as a framework for this diverse international effort to conserve irreplaceable germplasm and secure food for humanity indefinitely (Fowler, 2008).

Long-term plant germplasm conservation is a multi-phase, multi-generational operation accompanied by daunting biological and logistical challenges intractable to ready solutions (Lusty et al., 2021a; Engels and Ebert, 2021b; Gepts, 2023). For example, sustaining genebank operations over the long term is problematic when long-term financial investments have not been secured for that mission (Fu, 2017; Engels and Ebert, 2021b). Genetic drift and non-random viability selection from germplasm maintenance and regeneration (i.e., propagation) can lead to genetic erosion and vulnerability in germplasm of selfing and outcrossing plants (FAO, 2010; Westengen et al., 2013; Brown and Hodgkin, 2015; Khoury et al., 2022; Fu et al., 2023). Given those challenges, one could reasonably question whether the maintenance of genetic integrity of conserved accessions following decades or even centuries of *ex situ* conservation in genebanks is an achievable goal.

Maintaining plant germplasm in cold storage, tissue culture, or as growing plants in the field or greenhouse are considered to be the most cost-effective conservation strategies, particularly for orthodox seeds (Li and Pritchard, 2009). Thus, a seed genebank was first developed to store seeds in standard cold and dry conditions as a means to conserve the genetic diversity and identity of a sample (Frankel and Soulé, 1981), followed by the establishment of field genebanks, *in vitro* genebanks and cryobanks. Through time, these conservation approaches were refined, and guidelines emerged for optimal methods, as summarized in the Genebank Standards for Plant Genetic Resources for Food and Agriculture in 1994 for seed genebanks (FAO, 1994), followed by revision and expansion in 2014 (FAO, 2014) and subsequent practical guides (FAO, 2022a, b, c). Implementing the preceding technical approaches has proven challenging for genebanks (e.g., Hawkes et al., 2001; Hammer, 2004; Engelmann and Rao, 2012; Dulloo et al., 2013; Díez et al., 2018; Engels and Ebert, 2021b; Hay et al., 2021; Lusty et al., 2021b). Notably, many plant germplasm accessions have not been adequately regenerated, leading to regeneration backlogs in many genebanks (FAO, 2010, 2016). Some viability monitoring procedures were found to be less effective than expected in seven genebanks of the Consultative Group for International Agricultural Research (CGIAR) (Hay et al., 2021). Other logistical challenges have complicated germplasm management efficiency (Díez et al., 2018; Lusty et al., 2021b; Engels and Ebert, 2024).

In this paper, we will address the simplified question: will a plant germplasm accession conserved in a genebank change genetically over long-term conservation? Specifically, we will highlight the challenges in *ex situ* germplasm conservation, explore the expected answer from the joint actions of evolutionary driving forces associated with genebank operations, search for the empirical evidence for genetic changes over time from literature, and reason the significance of the answer for long-term germplasm management and conservation. Our motivation here is to draw attention to the importance of understanding the evolutionary dynamics and expectations of long-term germplasm conservation (Frankel, 1974; Schaal and Leverich, 2004) for effective genebank operations.

## 2 Challenges in *ex situ* germplasm conservation

Currently, 2000 genebanks worldwide conserve over 7.4 million plant germplasm accessions of more than 16,500 plant species and about two million accessions are estimated to be unique (FAO, 2010; Engels and Ebert, 2024). Over the years of conservation, it has been gradually realized that such a large-scale long-term germplasm conservation was not purposefully, nor effectively, designed at the beginning (Pistorius, 1997; Engels and Ebert, 2021a). Consequently, this global conservation effort has evolved to become a complicated and challenging endeavor (Zohrabian, 1995; Imperial College Wye, 2002; Engels, 2004; Perrino, 2005; Qualset and Shands, 2005; Díez et al., 2018; Engels and Ebert, 2021b; Lusty et al., 2021a). Here, we highlight some of the most important challenges to the international plant germplasm system and plant germplasm management which can contribute to the genetic changes of conserved germplasm.

### 2.1 Genebank system is underfunded

A genebank comprises essential infrastructure for short- and long-term seed storage and clonally-propagated germplasm on farms and successful germplasm management operations encompassing safety backup, regeneration and characterization, germplasm distribution, and data management (Engels and Visser, 2003). It requires adequate funding for staffing, infrastructure for information technology (IT) systems, and applied research to develop technologies to improve genebank operations. Fu (2017) identified the 10 most critical vulnerabilities to plant germplasm genebanks around the world. Specifically, these genebanks are generally under stress, largely from inadequate public investment, weakened political support, and insufficient stakeholder engagement. The insufficient support has impacted every type of genebank operation, and many elements of applied research focused on plant germplasm management are also diminishing. These vulnerabilities, along with those recently reported (Díez et al., 2018; Lusty et al., 2021a; Herbold and Engels, 2023) and other assessments (e.g., Gepts, 2023), clearly indicate that genebanks worldwide are generally underfunded and not sustainable over the long term, unless sufficient funding support is secured. For example, the actual operation budget for the USDA-ARS National Plant Germplasm System (NPGS) increased from US$20.4 million in 1994 (Clark et al., 1997) to $54.5 million in 2023 (https://www.ars-grin.gov/Pages/NPGS; accessed on 11 August 2024), but the true budget increase over the 30 years was only 16.2% when the agricultural research deflator was taken into account. The number of accessions in the NPGS increased 39.1% (from 445,879 accessions in 1994 to 620,254 accessions in 2024). However, the NPGS operation budget, when adjusted to 1999 dollars, actually decreased from the peak of approximately $38 million in 2003 down to $31 million in 2023 (Gayle Volk, personal communication, 2024).

Plant germplasm can be conserved in the form of seeds, plants under cultivation in the field or greenhouses, *in vitro* slow growth tissue culture, dormant buds and shoot tips that are cryopreserved, and pollen. Each genebank requires specific conservation facilities to conserve various forms of plant germplasm and has its own challenges for developing and implementing different conservation protocols and technologies. For example, managing germplasm as tissue culture requires the development of specific *in vitro* protocols incorporating different factors such as temperature, light conditions, growth medium composition, and the presence/absence of plant growth regulators and inhibitors (e.g., Benelli et al., 2022). Different methods and techniques are needed for cryopreservation of plant tissues from different species (Jiroutová and Sedlák, 2020) and to expand the number of cryostored accessions to safeguard greater genetic diversity (Chaudhury and Malik, 2016; Jenderek and Reed, 2017). The developed protocols are primarily effective only for germplasm of specific species, requiring more applied research that is typically under-supported.

### 2.2 Genebank management is not adaptive to declining resources

The major goal of conserving plant germplasm in genebanks is to ensure that crop germplasm and their wild relatives are available for current and future use by farmers, plant breeders, and researchers (Engels and Ebert, 2024). To achieve such a goal, many operational procedures are developed to manage plant germplasm; plant samples are monitored for viability; samples are regenerated to ‘refresh’ the slowly deteriorating stock or multiplied to increase the conserved stock. To facilitate germplasm use, the genebank operations also include germplasm evaluation, characterization, and documentation. A germplasm information system, such as GRIN-Global (https://www.grin-global.org/; accessed on 11 August 2024), is required to provide researchers and breeders with data associated with accessions and to enable requests for access to that germplasm. To enhance genebank management, FAO developed the voluntary Genebank Standards for Plant Genetic Resources for Food and Agriculture for three types of genebanks (FAO, 2014) and practical guides (FAO, 2022a, b, c). However, concerns are not lacking about these practical guides (Engels and Ebert, 2021b; Hay et al., 2021; Lusty et al., 2021b), as inconsistent application of the operation procedures for accession monitoring that involves collecting, managing, and analyzing viability data was found in seven CGIAR genebanks (Hay et al., 2021).

The main problem is that genebank management is not adaptive to insufficient funding and declining resources, although some commendable effort was reported in recent years to explore a monitoring system for the effectiveness of operations across all CGIAR genebanks (Lusty et al., 2021b). Given the declining funding and resources, genebank operations should have evolved by prioritizing genebank activities around genebank maintenance and germplasm regeneration to prevent germplasm loss (Fu, 2017). Germplasm regeneration and seed viability tests are two critical procedures required to maintain the long-term survival of germplasm propagated by seeds (Breese, 1989; Sackville Hamilton and Chorlton, 1997; Lawrence, 2002), and these operations are costly, requiring laboratory, land, labour, material and supplies, and complicated planning (Koo et al., 2004; Schreinemachers et al., 2014; Anonymous, 2020). For example, regenerating the whole collection of 120,302 accessions at Plant Gene Resources of Canada would require up to 34 years of continuous investment with an annual resource-allowable regeneration capacity of 3500 accessions (Diederichsen and Davidson, 2022). Thus, it is not surprising that accession regeneration backlogs exist and will continue to exist in many genebanks, if genebank management is not adaptive for practically achievable conservation goals (Engels and Rao, 1998; Imperial College Wye, 2002; Fowler and Hodgkin, 2004; Hammer, 2004; Qualset and Shands, 2005; Dulloo et al., 2008). The FAO surveys indicated that 37% of the surveyed genebanks reported regeneration backlogs in 2010 (FAO, 2010) and that 5.7% of the reported collections were regenerated in 2014 and additional 137,000 accessions needed regeneration (FAO, 2016). An insufficient rate of germplasm regeneration was also reported as one of the seven major operational challenges for the Spanish Plant Genetic Resources Network (Díez et al., 2018). Similarly, extensive backlogs have recently been identified as the most severe operational challenge for the USDA-ARS NPGS (https://www.ars-grin.gov/Pages/NPGS; accessed on 11 August 2024), requiring significant additional funding support as evaluated in a report of the USDA-ARS National Genetic Resources Advisory Council (https://nareeeab.ree.usda.gov/sites/default/files/2023-11/NGRAC_NPGS_Strategic_Plan_Report_FINAL_APPROVAL.pdf; accessed on 31 August 2024). The unfortunate fact, however, is that the extent of current regeneration backlogs and subsequent germplasm loss across the world’s genebanks remains unknown.

Viability testing is required to ascertain whether conserved seeds maintain sufficient viability during storage (e.g., van Treuren et al., 2013, 2018). Nonetheless, the historical viability monitoring data from seven CGIAR genebanks (Hay et al., 2021) revealed several management issues for viability monitoring, including an inadequate information management system with data errors and loss, inadequate viability test procedures with insufficient data recorded, insufficient value of the collected viability data to quantify seed longevity under long-term genebank storage conditions, inadequate application of viability data to inform management decisions, and difficulties in conducting viability tests of germplasm with insufficient seed lots, identity and phytosanitary requirements. These issues clearly demonstrated that the key genebank operation such as viability monitoring was not optimized, nor evolved, for its function to serve germplasm conservation effectively.

## 3 Genetic changes are expected in conserved germplasm

As mentioned above, good genebank management encompasses many operational procedures from germplasm acquisition for storage to distribution for use. These genebank operations, particularly for germplasm monitoring and regeneration, will repeat for many generations over the long term, during which genetic changes will occur in conserved germplasm, as explained below.

### 3.1 Genetic changes from evolutionary forces in a genebank system

An accession is the basic unit of germplasm conserved in a genebank and consists of germplasm samples collected from one location or source at a specific time. The samples comprise plant propagules, including whole plants, seeds, pollen, or other plant organs and tissue. These plant propagules conserved in a genebank will lose vigor or viability eventually and need to be regenerated. The newly regenerated propagules will represent the original accession and may have their genetic profiles changed either fractionally or substantially. From the perspective of population genetics (e.g., see Hedrick, 2010), this germplasm accession can be considered a finite population of plant propagules subject to four evolutionary forces: selection, mutation, genetic drift, and gene flow operating in the different genebank environments. The unique feature of conserved germplasm is the environment in which the germplasm is conserved: a cold environment for storage and a field or natural environment for accession regeneration. These environments may differ from those for a natural plant population and among three types of genebanks, but the four evolutionary forces can still operate with different strengths and directions on the accession over the different stages of conservation. Also, a small effective population size would introduce the effect of a genetic bottleneck for the conserved accession and can jointly increase the effects of genetic drift and inbreeding. In a population of size 100 or smaller, the allelic diversity that can be maintained over time can fluctuate greatly (Kimura and Crow, 1964). Alleles can be more readily lost or fixed compared to allelic diversity in a larger population (Hedrick, 2010; Der et al., 2011; Molina and Earn, 2018). Thus, genetic changes in the accession over time are expected to occur under these genebank conditions.

**Figure 1:**
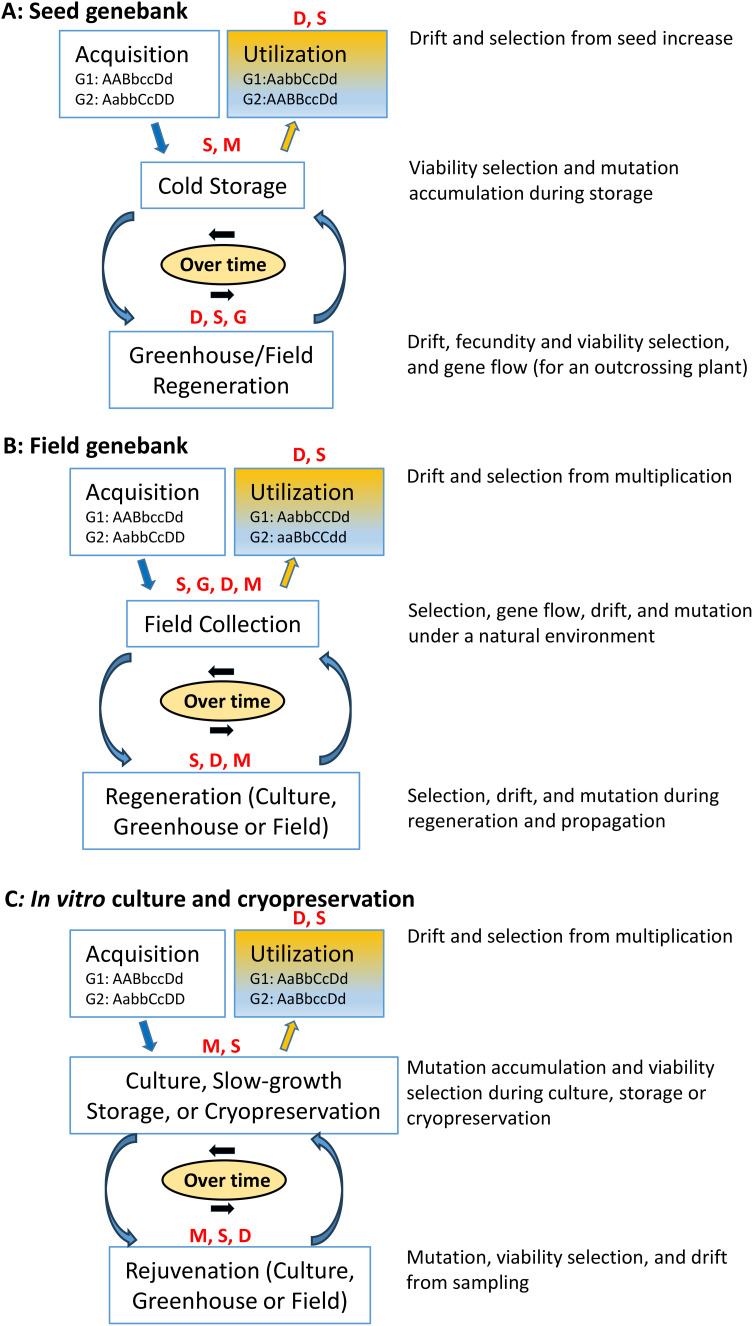
Illustration of the genetic changes of a conserved accession (with two initial genotypes at four diallelic loci: G1: AABbccDd and G2: AabbCcDD) from acquisition, conservation to utilization over time in each of the three genebanks (**A**: seed genebank, **B**: field genebank, and **C**: *in vitro* culture and cryopreservation) with its four major operation stages subject to the joint actions of evolutionary forces [selection (S), mutation (M), genetic drift (D) and gene flow (G)].

To understand the genetic changes of an accession resulting from evolutionary forces, we consider three types of *ex situ* germplasm collections and illustrate four evolutionary forces that could potentially act jointly on an accession during long-term conservation, as in **Figure 1**. For germplasm accessions conserved as seeds, four evolutionary forces can jointly act on the accession in every element of germplasm management, from acquisition to cold storage, regeneration, through utilization (**Figure 1A**). For example, the accession can be subject to viability selection (from germplasm viability loss) and mutation during cold storage, and subject to genetic drift, fecundity and viability selection, and gene flow (for an outcrossing plant) in greenhouse and/or field regeneration. During long-term conservation, many cycles of storage and regeneration can occur, genotypes comprising the accession will change from joint evolutionary forces, and some alleles will be fixed or lost before the utilization stage, as illustrated with two initial genotypes at four diallelic loci (G1: AABbccDd and G2: AabbCcDD) in **Figure 1A**. For example, these two original genotypes were changed over time with the fixation from the allele B to b of G1:AabbCcDd and the loss from the allele b to B of G2:AABBccDd before the stage of germplasm use (**Figure 1A**).

Expectations for genetic changes in the germplasm conserved in the field and *in vitro* culture resemble those discussed above (**Figures 1B, C**). Several evolutionary forces will act on the genotypes of the accession during conservation stages, and genetic changes will occur during long-term conservation. For example, over time, the two original genotypes (G1: AABbccDd and G2: AabbCcDD) were changed with the loss of the allele c of G1 and allele D of G2 and the fixation of the allele b of G1 and allele C of G2 before the use stage (**Figure 1B**). The differences expected among the three types of germplasm collections can result from different strengths of evolutionary forces acting on the conserved genotypes of different germplasm types with variable effects under different conservation environments. For example, point mutations might be more consequential in genotypes under cold storage, whereas *in vitro* culture changes in longer tracts of the genome, sometimes termed somatic mutations, might occur. Stronger viability selection would be imposed on the genotypes under long-term cold storage than those under short-term field conditions. Genetic drift and genetic bottlenecks can occur from germplasm sampling within an accession of a finite size. The gene flow could also occur on the germplasm of an outcrossing species during field regenerations and from the inadvertent mixing of seed germplasm.

There are some unique features of genetic changes expected for the germplasm conserved as accessions in genebanks. First, the evolutionary forces operating in a small population, like a typical accession, will jointly act more strongly than those in a large natural population. Second, long-term conservation can require cycles of storage and regeneration depending on germplasm biology, and the cumulative impacts of each evolutionary force, particularly mutation, will be stronger than those in the natural population during a few generations. Jain (1961) investigated the loss of genetic diversity from 19 generations of bulk composite crossing in annual self-pollinating cereals and found that 50–70% of the variation for height and heading was lost and that significant loss of genetic diversity could be detected already after just 10 generations. Third, the impact of each evolutionary force on conserved germplasm can vary greatly among the three types of genebanks. For example, somatic mutation will predominantly contribute to the genetic changes of conserved clonal explants, whereas selection will be dominant in the germplasm of a field genebank. However, these are simplified versions for illustrations of possible genetic changes expected under long-term germplasm conservation in different genebanks. Many relevant conservation conditions, such as storage temperature and relative humidity, and seed biology, can also jointly influence the rate of genetic changes in conserved germplasm, together with those practical constraints in genebank operations.

To our knowledge, these evolutionary dynamics and expectations of long-term germplasm conservation are not unknown (Schoen and Brown, 2001), as abundant literature exists on plant conservation genetics (e.g., see Henry, 2006; Kramer and Havens, 2009; Sgrò et al., 2011), even in *ex-situ* germplasm conservation (e.g., Frankel, 1974; Cross and Wallace, 1994; Schoen et al., 1998; Schaal and Leverich, 2004; Richards et al., 2010), yet they do not receive sufficient attention in routine genebank operations. For example, Cross and Wallace (1994) predicted through computer simulations and mathematic modeling that the loss of genetic diversity within heterogeneous self-pollinating genebank accessions is severe after several regeneration cycles. This prediction is compatible with those by Richards et al. (2010) when considering additional genebank operation procedures. Schoen et al. (1998) modeled the accumulation of mildly deleterious mutations accompanying recurrent regeneration of plant germplasm under regeneration conditions and suggested that mutation accumulation has the potential to reduce the viability of germplasm managed *ex situ*. As described above, these theoretical studies provided the same expectations on genetic changes from some operational procedures, such as germplasm regeneration.

### 3.2 Genetic changes caused by the constraints of germplasm management

Many factors in germplasm management can directly and/or indirectly impact the biology and viability of conserved germplasm and, consequently, contribute to germplasm genetic changes over long-term conservation. The influencing factors could come from the deteriorating conditions and/or declining sustainability of a genebank, including essential genebank infrastructure for storage conditions and capacity, IT infrastructure for information systems, genebank funding for staffing, and supportive research to develop technologies. As mentioned above, issues affecting genebank conditions and sustainability are not lacking (Fu, 2017; Díez et al., 2018; Engels and Ebert, 2021b; Hay et al., 2021; Lusty et al., 2021b), and consequently, conserved germplasm can be vulnerable, or even lost (FAO, 2010). An alarming example of germplasm vulnerability is the pressing need of regenerating and/or repropagating 98,842 accessions in 2023 and other 78,125 accessions within five years as reported in the USDA-ARS NPGS plan (https://www.ars-grin.gov/Pages/NPGS; accessed on 11 August 2024).

Many genebank operational procedures, as described in the practical guides of the Genebank Standards (FAO, 2022a, b, c), can also contribute to genetic changes in conserved germplasm. **Table 1** lists the relevant genebank operational procedures, their management impacts, and possible evolutionary forces acting on conserved germplasm. For example, seed increase after germplasm acquisition for storage either in a greenhouse or field could change the genetic make-up of an accession through the effects of genetic drift and artificial selection. Improper planting practices in a field genebank can generate increased viability selection on the conserved germplasm. Accession multiplication in an *in vitro* genebank can result in genetic changes via genetic drift and somatic mutation. These operational procedures can individually and/or jointly influence the genetics of the conserved germplasm through various evolutionary forces at different stages of conservation, and genetic changes can be accumulated over long-term conservation.

**Table 1:** List of relevant genebank operational procedures, management impacts and possible evolutionary forces acting on conserved germplasm.

Genebank operation procedure	Management impact	Possible evolutionary forces acting on germplasm
Seed genebank		
Germplasm acquisition	Accession identity	
Seed increase for storage (GH or Field)	Accession amount and vigor	Genetic drift and artificial selection
Cleaning and drying	Germplasm health	Artificial selection
Storage control	Germplasm long-term storage	Mutation and viability selection
Routine germination testing	Germplasm longevity	Viability selection
Regeneration (GH or Field)	Germplasm revitalization	Genetic drift, fecundity and viability selection, and gene flow for OG
Seed increase for use (GH or Field)	Germplasm availability	Genetic drift and artificial selection
Field genebank		
Germplasm acquisition	Accession identity	
Field preparation	Germplasm field planting	Viability and fecudity selection
Appropriate planting practice	Accession growth	Viability selection
Field management	Accession growth and survival	Fecudity and viability selection, gene flow, genetic drift, and mutation
Regular germplasm monitoring	Accession survival	Viability selection
Regeneration and propagation	Germplasm revitalization	Artificial selection, somatic mutation, and genetic drift
True-to-type verification	Accession identity	Somatic mutation
Germplasm increase for use (GH or Field)	Germplasm availability	Genetic drift and artificial selection
In vitro culture genebank		
Germplasm acquisition	Accession identity	
Apply in vitro culture technique	Accession survival	Viability selection
Accession multiplication	Germplasm availability	Genetic drift and somatic mutation
Apply slow-growth storage	Accession growth and survival	Viability selection and mutation
And/or cryopreservation	Germplasm long-term storage	Viability selection and mutation
Regular in vitro culture monitoring	Accession survival	Viability selection
Appropriate recycling practices	Accession survival	Artificial selection and somatic mutation
Regeneration (GH or Field)	Germplasm revitalization	Somatic mutation, genetic drift, and viability selection
Germplasm increase for use (GH or Field)	Germplasm availability	Genetic drift and artificial selection

The listed procedures were extracted from the Practical Guide for the Application of the Genebank Standards for Plant Genetic Resources for Food and Agriculture (FAO, 2022a, b, c). GH, greenhouse and OG, outcrossing germplasm.

Recognizing the possible genetic impacts of an operational procedure would enhance the effectiveness of germplasm management, but it should also be noted that not all of the genebank issues and operational procedures can effectively be managed and/or controlled to avoid and/or minimize their genetic impacts. For example, a genebank facility with −18°C storage reflects an important long-term investment, and expanding the existing storage capacity, if existing space is fully used, may not always be feasible in the short term, generating constraints in germplasm storage and thus affecting germplasm viability. Developing a new and more effective *in vitro* storage protocol for existing germplasm is always desirable but could be too expensive to achieve, creating operational constraints for conserving *in vitro* germplasm. Both genebank and operational constraints can generate genetic impacts on conserved germplasm, as illustrated below.

In a seed genebank, regeneration is the operation of planting an aged accession when its viability has declined below a specified limit (or usually 85% viability), to obtain a fresh sample of highly viable seed in large quantity. As illustrated above, this procedure can generate genetic changes by genetic drift alone. Brown et al. (1997) illustrated the geometric power of recurrent regeneration for a single genetically heterogeneous accession in a collection with the formula: $S_{r}=3^{r}/p_{\text{o}}$ , where *S* is the required sample size, *r* is the number of regenerations, and *p*_o_ is the allelic frequency in the original accession. The formula allows the calculation of the sample size (in terms of the number of random gametes) *S*_i_ in the ith generation (*i*=0 to *r*) required to include at least one copy of more than 95% of alleles present in the original population with frequency greater than *p*_o_. **Table 2** lists the required sample sizes for variable levels of the original allele frequency po (0.3 to 0.005) with *r*=1 to 5 cycles of regeneration. When *p*_o_=0.05, the required sample size in the 1st regeneration is 60 seeds, but increases to 540 seeds in the 3rd regeneration, and further to 4860 seeds in the 5th regeneration. It is difficult in an actual genebank operation to achieve such a large sample size of 540 seeds for an accession of some plant species after the 3rd regeneration. Similarly, when *p*_o_ is smaller (say *p_o_* =0.01), it is practically impossible to achieve the required sample size of 900 seeds in the 2nd regeneration to keep the original allele frequency constant at 0.01.

**Table 2:** Possible practical limits (shown in brown), in a sample size larger than 300, that are required to include at least one copy of more than 95% of alleles present in the initial accession with a frequency greater than *p*_o_ over the cycles of regenerating a heterogeneous accession.

*p_0_*	The number of regeneration cycles				
	1	2	3	4	5
0.3	10	30	90	270	810
0.2	15	45	135	405	1215
0.1	30	90	270	810	2430
0.05	60	180	540	1620	4860
0.04	75	225	675	2025	6075
0,03	100	300	900	2700	8100
0.02	150	450	1350	4050	12150
0.01	300	900	2700	8100	24300
0.005	600	1800	5400	16200	48600

A similar example of an operational procedure such as regeneration imposing a constraint to maintain a trait during a regeneration cycle is given in **Table 3**. Sedcole (1977) presented several methods to determine the number of plants (or sample size; *S*) needed, with a specified high probability (*p*), to recover a given number of plants (*n*) possessing a trait, given that the trait occurs with a known probability (*q*) in the population. **Table 3** was generated with a custom R script based on Method I of Sedcole (1977) for variable levels of *q* and *n*, assuming *p*=0.95. When *q*=0.05, the sample size is 59 plants necessary to recover one plant with the trait and 311 plants to recover 10 plants with the trait. When *q*=0.01, the sample size is 299 plants necessary to recover one plant with the trait and 473 plants to find two plants with the trait. It has become clear that a large sample size is needed to recover a few plants with a trait of low frequency. Thus, it is generally difficult to achieve such a large sample size to maintain a trait in a field genebank. This example, along with those in **Table 2**, demonstrates that genebank operational procedures can impose constraints on the theoretical sample size required to keep genetic variation unchanged over the long term.

**Table 3:** Possible practical limits (shown in brown), in a sample size larger than 300, that are required to be 95% certain to recover a minimum number of plants with a trait of frequency *q* in an accession.

*q*	The minimum number of plants to be recovered								
	1	2	3	4	5	10	15	30	50
0.2	14	22	30	37	44	76	106	193	304
0.1	29	46	61	76	89	154	215	391	615
0.05	59	93	124	153	181	311	434	786	1237
0.04	74	117	156	192	227	390	544	984	1548
0.03	99	157	208	257	303	521	726	1313	2066
0.02	149	236	313	386	456	782	1091	1972	3102
0.01	299	473	628	773	913	1568	2185	3949	6211
0.005	598	947	1258	1549	1829	3138	4374	7903	12428

## 4 Empirical evidence for genetic changes in conserved germplasm

Concern was expressed about genetic changes of plant germplasm under conservation practices in the 1970s and 1980s (Roberts, 1973; Roos, 1980). Mutation may occur during storage (Ashton, 1956; Roberts, 1978) and selection could reduce the existing genetic variability by differential viability during seed storage and by differential productivity during seed regeneration (Roos, 1984a). Since then, empirical evidence for genetic changes in conserved germplasm has accumulated. Here, we highlight three lines of evidence.

### 4.1 Mutational changes under germplasm storage

Early studies have demonstrated that mutation occurred in seeds under storage (Ashton, 1956; Barton, 1961; Roberts, 1978; Roos, 1982; Dourado and Roberts, 1984). For example, Dourado and Roberts reported (1) that pea seeds stored at 35°C and 16.5% moisture content for 40 and 57 days had a mutation frequency (percentage of seeds containing recessive point mutations) of 3% to 4% and (2) that barley seeds at 15.5% moisture content stored at 50°C for 42 and 54 h, and at 35°C for 28 and 39 days had a mutation frequency that increased from zero to between about 0.3% and 0.9%. While the seed storage conditions in the early studies differed significantly from those currently used in most genebanks, the findings of mutation occurrence are still relevant to current genebank operations for understanding the genetic changes of conserved germplasm (Ashton, 1956; Barton, 1961; Schoen et al., 1998).

Recently, we conducted the first mutation investigation unique to plant germplasm conserved *ex situ* worldwide (Fu et al., 2023). Specifically, RNA-Seq technology was applied to sequence 490 individual plants representing the germplasm collections of barley, wheat, oat, soybean, maize, rapa (*Brassica rapa* L.), and sunflower that were conserved in Plant Gene Resources of Canada. Deleterious genetic variants were detected in extremely constrained genic regions based on the scores of both Sorting Intolerant From Tolerant (Vaser et al., 2015) and Genomic Evolutionary Rate Profiling (Davydov et al., 2010). Mutational changes with respect to contrasting germplasm storage years and regeneration numbers are illustrated in **Table 4**. Germplasm stored longer since the last germplasm regeneration showed an increase in the proportion of the deleterious SNPs (dSNPs) over all the detected SNPs in five collections (barley, wheat, maize, rapa, and sunflower) and a decrease in two collections (oat and soybean). Note that the differences in storage year ranged from 5 to 22 for the seven collections. Similarly, barley and sunflower germplasm stored longer showed an increase in the proportion of fixed dSNPs, while oat, maize, and rapa germplasm stored longer displayed a decrease in fixed dSNPs. For germplasm with one more regeneration, an increase in the proportion of dSNPs was found in three collections (barley, soybean, and sunflower) and a decrease in four collections (wheat, oat, maize, and rapa). Similarly, soybean, maize, and rapa germplasm with one more regeneration displayed an increase in the proportion of fixed dSNPs, while barley, wheat, and sunflower germplasm with one more regeneration displayed a decrease in fixed dSNPs. These findings clearly demonstrate that mutational changes occurred in the assayed germplasm conserved in the genebank over the last 50 years. More collections showed an increase in dSNPs from the longer germplasm storage than the collections with decreased dSNPs. Similarly, more collections showed a decrease in dSNPs from more germplasm regeneration than the collections with increased dSNPs.

**Table 4:** Estimates of mutational changes in paired groups of randomly selected accessions with different storage years and numbers of germplasm regeneration in seven germplasm collections (barley, wheat, oat, soybean, maize, rapa, and sunflower).

Storage year group	Barley		Wheat		Oat		Soybean		Maize		Rapa		Sunflower	
	10Y	27Y	20Y	25Y	16Y	21Y	10Y	20Y	7Y	29Y	5Y	16Y	6Y	27Y
Sample size	16	16	12	12	10	10	15	15	18	18	20	20	15	15
dSNP proportion														
Estimate	112.5	116.9	331.8	358.7	2083.4	1586.5	138.6	134.0	6.5	7.1	115.7	136.8	75.3	78.8
Standard deviation	1.3	1.7	3.1	5.2	36.4	264.8	2.1	2.0	0.1	0.1	1.5	1.4	0.6	0.8
Difference	4.4		26.9		-496.9		-4.7		0.6		21.2		3.5	
*P* value	***		***		**		***		***		***		***	
Fixed dSNP proportion														
Estimate	8.5	11.9	23.0	23.3	49.9	34.6	8.0	7.8	0.4	0.2	4.2	3.5	3.9	5.2
Standard deviation	0.5	0.6	1.2	0.8	2.4	4.2	0.5	0.3	0.0	0.1	0.1	0.1	0.2	0.3
Difference	3.4		0.3		-15.3		-0.2		-0.2		-0.7		1.3	
*P* value	***				***				***		***		***	

Regeneration group	Barley		Wheat		Oat		Soybean		Maize		Rapa		Sunflower	
	RF1	RF2	RF1	RF2	RF1	RF2	RF1	RF2	RF1	RF2	RF1	RF2	RF1	RF2
Sample size	24	24	18	18	9	9	14	14	18	18	15	15	27	27
dSNP proportion														
Estimate	115.5	117.8	343.9	324.9	2275.7	2043.8	136.7	150.5	7.0	6.9	122.9	121.4	70.9	76.1
Standard deviation	0.9	1.0	2.6	3.5	56.0	81.9	1.4	3.1	0.1	0.1	1.0	0.7	0.5	0.5
Difference	2.2		-19.0		-231.9		13.8		-0.2		-1.5		5.2	
*P* value	***		***		***		***		*		**		***	
Fixed dSNP proportion														
Estimate	7.0	6.1	18.1	17.2	50.7	53.5	7.3	9.6	0.2	0.7	4.8	5.5	3.2	2.7
Standard deviation	0.3	0.2	0.7	1.0	3.4	3.5	0.5	0.5	0.0	0.1	0.2	0.2	0.1	0.1
Difference	-0.8		-0.8		2.8		2.3		0.4		0.6		-0.5	
*P* value	***		*				***		***		***		***	

The storage year group label shows the years of storage after the last germplasm regeneration (e.g., 10Y and 27Y=10 and 27 years after the last regeneration, respectively) and the regeneration group label shows the number of germplasm regenerations since the accession acquisition (e.g., RF1 and RF2=the 1^st^ and 2^nd^ regenerations, respectively). The proportions of the deleterious or fixed deleterious SNPs over all the SNPs detected (dSNP or fixed dSNP proportions, respectively) are enlarged to a 100,000 scale for ease of comparison. The significance levels of an *F*-test for a difference at 0.05, 0.01, and 0.001 are shown with *P* values of *, **, and ***, respectively. The data is acquired from Fu et al. (2023) and re-tabulated for illustration.

Somatic variation has been long known to occur in long-term *in vitro* tissue cultures with unintended genetic and/or epigenetic changes (Phillips et al., 1994; Bednarek et al., 2021) and *in vitro* conservation of plant tissues is dependent on *in vitro* tissue culture techniques (FAO, 2014). *In vitro* somatic mutation can contribute to the production of novel genotypes of ornamental plants and is a source of useful new traits for both production and biotic resistance (Leva and Rinaldi, 2017), but it can also pose challenges in conserving plant regenerates over long culture periods (Reed et al., 2004; Pence, 2010). Any plant regenerates observed with somatic variation in *in vitro* operational procedures are treated as off-type and discarded, as such variation can compromise clone identity (FAO, 2022c).

### 4.2 Genetic shift in conserved germplasm

Gene loss and significant shift in maturity were observed in the last generations of six different pearl millet germplasm pools that were created with an equal mixture of elite inbreds and introductions and advanced in isolation three to five generations (Burton, 1976). Roos conducted an artificial aging study to investigate the potential genetic shifts in mixed bean populations following long-term seed storage and regeneration, which demonstrated the potential for loss in genetic viability within heterogeneous germplasm accessions during long-term storage (Roos, 1984a), and predicted that only two out of eight bean cultivars would survive 15 cycles of regeneration (Roos, 1984b). Chebotar et al. (2003) reported that the significant shift of microsatellite (SSR) alleles in rye accession R 52 was likely associated with the directional selection (or possibly caused by the low seed survival) imposed from strong winter damage in the 1971, 1979, and 1983 regenerations. McLean-Rodríguez et al. (2021) applied genotyping-by-sequencing technology to analyze 13 maize accessions that originated from the state of Morelos, Mexico, conserved *ex situ* since 1967 and retrieved from the farms of the same donor families in 2017, and found evidence of directional selection in specific loci in the on-farm samples. This evidence was consistent with farmers’ ear-based mass selection criteria. Similarly, several other studies comparing *in situ* populations to *ex situ* germplasm generally found significant genetic shifts in *in situ* populations (Tapia and Estrella, 2001; Šuštar-Vozlič et al., 2004; Shewayrga et al., 2008; Sun et al., 2012). Given both *in situ* and *ex situ* populations are changing over time, it is difficult to assess the degree to which changes are due to genebank practices associated with the *ex situ* accessions versus the evolution of *in situ* populations (Camadro, 2012). Despite this caveat, artificial or natural selection clearly contributed to the genetic shifts revealed in conserved germplasm. However, there are no reports yet on the genetic shifts in plant germplasm stored in cryopreservation facilities.

### 4.3 Genetic diversity changes in conserved germplasm

Over the last three decades, studies have been made on the genetic changes within and among conserved accessions through genetic or phenotypic comparisons of diversity changes. In the search for empirical evidence for genetic erosion in *ex situ* germplasm conservation, Khoury et al. (2022) found 26 articles published between 1995 and 2019. These articles documented genetic changes over time that were largely from germplasm regeneration, viability decline, and maintenance activities. Specifically, more than 88% of the articles had evidence of loss or disappearance, and 30% (also) had evidence of an increase or appearance of new diversity over time. Based on the acquired evidence, Khoury et al. concluded that “genetic change and genetic erosion of samples in genebanks appear to be the rule rather than the exception.”

Here, we highlight some studies to illustrate the evidence for genetic diversity changes in conserved germplasm after multiple regenerations in seed genebanks. Parzies et al. (2000) employed morphological and isozyme markers to analyze two barley landraces from Syria that had been stored for 10, 40, and 72 years and found significant declines in average gene diversity, alleles per locus and percentage polymorphic loci and increase in genetic differentiation among accessions over the time of storage. The observed genetic changes were due to multiple rejuvenations, on average every 5.3 years. Chebotar et al. (2003) employed SSR markers to analyze six outcrossing rye accessions conserved at the Institute for Plant Genetic and Crop Plant Research at Gatersleben, Germany, and found that four assayed accessions with 7 to 13 regeneration cycles had significantly different allele frequencies. Cieslarová et al. (2012) applied SSR markers to analyze 10 pea accessions with up to eight regeneration cycles conserved in the Czech National Genebank and found significant differences in allele frequencies and genetic composition in six out of the 10 assayed accessions. Wen et al. (2011) used SNP markers to investigate the genetic integrity of 20 outcrossing maize landrace accessions with one or two regenerations in five maize genebanks and found that three studied accessions had significant changes in the average number of alleles per locus and 10 accessions displayed significantly different SNP allelic frequencies.

Most of the genetic diversity studies analyzed by Khoury et al. (2022) did not examine the genetic impacts of specific evolutionary forces, but genetic drift during germplasm multiplication and viability selection under storage could be reasoned to be the dominant factors. A few studies below were highlighted to show that genetic diversity changes are also associated with seed genebank practices. Chebotar et al. (2003) found the occurrence of novel SSR alleles in the last regenerated samples of five rye accessions. Similarly, the occurrence of novel SSR alleles was also found in the last regenerated samples of some self-compatible pea accessions (Cieslarová et al., 2012). The detection of novel alleles indicates that the regeneration practices employed previously for the assayed rye or pea germplasm may have had unintentional introgression or mixing from other samples. Steiner et al. (1997) applied storage-protein electrophoresis to assess the genetic purity of seven accessions of the Nürnberg oats of 1831 stored at Freising, Braunschweig, and Linz and found that these accessions had two novel electrophoretic phenotypes with frequencies of five and two. The genetic changes of a pure oat line were generated from various types of contaminations by human error in germplasm management, largely ranging from identity contamination by the respective other Nürnberg phenotype and/or foreign phenotypes up to the replacement of a line by a foreign phenotype.

## 5 Implications for long-term plant germplasm conservation

As discussed above, genetic changes in conserved germplasm will occur over long-term conservation. Recognizing this evolutionary principle of germplasm conservation is critical to achieve the key objective of genebanks: to support food security indefinitely. This evolutionary principle should play a role in the future research on conserved germplasm and the development of effective genebank operational procedures.

### 5.1 Evolutionary research on conserved germplasm

To inform long-term germplasm conservation requires knowledge about the evolutionary dynamics of conserved germplasm over time under variable genebank conditions. However, supportive germplasm research that is aimed at addressing genebank issues associated with long-term conservation is scarcer than generally thought (Fu, 2017; Engels and Ebert, 2021b). Many important questions about long-term germplasm conservation remain unexplored. These include but are not limited to (1) how much genetic composition change occurs in germplasm conserved under current, even optimal, storage conditions; (2) what is the extent of mutation accumulation over long-term storage and what is its genetic consequence on germplasm longevity; (3) how much impact does genetic drift have upon germplasm after multiple cycles of regenerating germplasm; and (4) how much genetic change is linked to germplasm adaptation to a new environment of genebank storage and germplasm regeneration. Answers to these questions will allow for a better understanding of how conserved germplasm evolves under genebank conditions, but need to be generated from more studies on the evolutionary changes in conserved germplasm. These studies can be pursued empirically and/or via AI-based computer simulations for better prediction of genetic changes. The resurrection approach of reviving ancestors from stored propagules and comparing them with descendants under common conditions (Franks et al., 2018; Spear et al., 2023) can be powerful for studying and predicting long-term genetic changes in conserved germplasm. Our mutation screening of conserved germplasm (Fu et al., 2023; Fu, 2024a, b) represented the first preliminary attempt in this direction.

### 5.2 Genebank standards updates

The voluntary Genebank Standards (FAO, 2014) have supported genebank operations to conserve germplasm worldwide. However, some recommendations of these standards are outdated, and related operational procedures were developed without sufficient consideration of evolutionary changes in long-term germplasm conservation. Their effectiveness for long-term germplasm conservation is questionable (Hay and Whitehouse, 2017; Engels and Ebert, 2021b, 2024; Hay et al., 2021; Lusty et al., 2021b; Bhattacharya and Mummenhoff, 2024). Here, we illustrate some caveats associated with the operational procedures for genetic integrity and germplasm regeneration. Maintenance of Genetic Integrity is one of the eight genebank principles developed in the Genebank Standards (FAO, 2014). However, the term genetic integrity is not defined. The principle serves as a goal only, but this goal is unachievable for long-term conservation, and consequently, the maintenance of genetic integrity over time is technically misleading. The genetic changes in conserved germplasm can be considered at the genome level as directional changes in responses to conservation environments and random changes from conservation practices. Genebank operation procedures can generate genetic changes from genetic drift, differential selection, and/or somatic mutation (**Table 1**), but such genetic changes will largely occur randomly across various chromosomes of a genome. These changes are not expected to significantly alter the genetic composition currently conserved for a given trait of future interest, and consequently, the original genetic diversity of an accession for the trait could be largely intact. For example, the average plant height of a selfing-plant accession may not necessarily change significantly over 10 cycles of field regenerations with only eight seeds used for each regeneration, as the strong genetic drift from the small sample size does not target the genetic composition of a specific trait such as plant height. Genetic responses to conservation environments, such as cold storage in genebanks or germplasm regeneration in fields, will occur and reflect the adaptive genetic changes from accumulative mutations of various natures (such as epigenetic mutations) and viability selection. This directional change will occur mainly at a few functional regions of the genome and may not necessarily introduce significant changes in the other genomic regions, so the original genetic diversity of an accession may not change much. However, the directional change will significantly affect germplasm survival under genebank conditions. Thus, it is more important to keep the conserved germplasm viable over the long term than to be concerned about its genetic changes. Following the same logic, one would argue that the genebank principle of Identity of Accessions is more important than Maintenance of Genetic Integrity, as identity loss or accession mislabelling (van de Wouw et al., 2011; Lusty et al., 2021b) will have more long-term impacts (van Hintum et al., 2011) than the genetic changes occurring to the accession. Also, it should be mentioned that existing adaptive genes in conserved germplasm are not necessarily adaptive to future environments, as illustrated with the selection against domestication alleles in introduced rabbit populations (Andrade et al., 2024) and that newly modified or mutated genes in conserved germplasm are not necessarily detrimental for its future adaptation to new environments. For example, some new mutations could be beneficial (Fu et al., 2019) to allow for adaptation to a new environment (Couce et al., 2024).

Germplasm regeneration is a critical, challenging, and costly component in genebank operation (Breese, 1989; Dulloo et al., 2008) and would be more beneficial to long-term conservation if the genebank principle of Germplasm Regeneration is developed to guide the related operations. As indicated above, multiple evolutionary forces can act jointly on germplasm regeneration and generate lasting biological and genetic impacts on conserved germplasm. Also, the long-term impact of germplasm regenerations is complicated and differs within and among germplasm of clonal, selfing, and outcrossing species, requiring informative guidance in the development of effective operational procedures. For example, the statement of the genebank activity, “Optimal regeneration procedures are used to minimize risk to the genetic integrity of the accession,” on page 32 of FAO (2022a), is technically not informative. The regeneration standards 4.4.2 and 5.5.2 for true-to-type verification (FAO, 2014) should be revised to “minimize the risk to accession identity,” not the genetic integrity of an accession. The advances in knowledge of germplasm regeneration have been reviewed and documented (Breese, 1989; Crossa, 1995; Sackville Hamilton and Chorlton, 1997; Lawrence, 2002; Engels and Visser, 2003; Dulloo et al., 2008; Redden and Partington, 2018), but such knowledge was not fully incorporated into the Genebank Standards. The genebank principle for Germplasm Regeneration can be developed based on germplasm biology to regenerate germplasm with minimized genetic changes through achievable operational procedures. The wisdom of ‘one size does not fit all’ is relevant here; separate standards and practical guides for regenerating germplasm of clonal, selfing, and outcrossing germplasm should be developed for relevant genebanks.

### 5.3 Darwinian genebank operations

Considering many uncontrollable factors such as the biological nature of germplasm (Lusty et al., 2021b), the challenges faced with current and future genebanks, and the practical constraints of germplasm management, one could conclude that genebank operations should be more adaptive, flexible, and feasible than before to deal with the genetic changes in conserved germplasm. First, genebank operations should evolve for practically achievable germplasm conservation in response to declining resources and, if needed, shift toward genebank priorities such as genebank maintenance and germplasm regeneration (Fu, 2017). Second, every operational procedure should consider the Genebank Standards for technical guidance, but also have a practically achievable goal to be developed toward minimizing (or technically delaying), not avoiding, genetic changes in conserved germplasm. Incorporation of the evolutionary rules into genebank operations (**Table 1**) will enhance the development of effective operational procedures and benefit the mission of long-term germplasm conservation. Third, each procedure needs to be regularly evaluated and modified accordingly for its effectiveness and efficiency with respect to the defined goal for different types of germplasm. Fourth, documentation is required on the effectiveness and efficiency of each applied procedure for better improvement of genebank operations and to allow for trackable analysis of genetic changes in conserved germplasm. For example, records should be made on the newly regenerated accessions with identified issues such as insufficient seeds, low germination, or damages of various nature, as large genetic changes are expected for these regenerated accessions.

To minimize the impact of genetic drift, germplasm regeneration should proceed, if practically feasible, with the acceptable sample size recommended for regenerating germplasm of 21 major food crops (Dulloo et al., 2008). However, these recommendations largely followed the rule of thumb developed by Crossa et al. (1993) to retain with a probability of 90 to 95% alleles with a frequency of 0.003 or higher, but regenerating germplasm of outcrossing species will require more seeds (Crossa, 1995). Different considerations may be required on the acceptable sample sizes of genetically heterogeneous versus homogenous accessions (Volk et al., 2021). If practically possible, time and frequency of germplasm regeneration should be optimized and minimized (Redden and Partington, 2018), respectively, and tools should be developed and implemented to prolong germplasm storage with sufficient viability (e.g., Volk et al., 2017), as frequent germplasm regenerations will accumulate more genetic impacts. Another explorable option is to develop an acceptable level of genetic changes in an accession for a given cycle or period of conservation under a genebank condition. However, deriving such an acceptable level requires knowledge about the extent and rate of genetic changes in conserved germplasm, which are currently lacking.

As genetic changes in conserved germplasm are inevitable and germplasm loss is preventable, another important management option is to consider the separation of viability selection from the other evolutionary forces in genebank operations (**Table 1**), as the former could generate genetic loss, while the latter will largely affect genetic changes. The separation of evolutionary forces would allow more weight to be placed on operational procedures subject to viability selection to avoid the loss of conserved germplasm and less on the operational procedures associated with the genetic changes, while maintaining the correct accession identity. To our knowledge, there are no formal reports published on the extent and nature of accession loss under long-term storage in genebanks over the last 60 years. Thus, the extent of genetic loss from nonviable germplasm under long-term storage, including the duplicated germplasm stored at the Svalbard Global Seed Vault, Norway, remains unknown. It is critical to enhance genebank operations with documentation and reports on germplasm loss of various types to develop effective measures to mitigate the loss of irreplaceable germplasm in genebanks.

## 6 Concluding remarks

The question of the genetic change of a conserved plant germplasm accession over time is relevant to the long-term effort of plant germplasm conservation. Its answer carries weight in future research on conserved germplasm and the development of genebank operational procedures to achieve the objectives of long-term germplasm conservation. It is expected that genetic changes of germplasm conserved over the long term under genebank conditions will occur commonly as an evolutionary rule, not as an exception. Incorporating evolutionary biology into the Genebank Standards and operational procedures will benefit the mission of long-term germplasm conservation.
